# Internal Medicine Residents’ Challenges in Trauma-Informed Care and Impact on Patient Care: A Multiple-Methods Study

**DOI:** 10.1007/s11606-026-10260-6

**Published:** 2026-03-16

**Authors:** Uma Thachapuzha, Muriel Jean-Jacques, Sarah Chuzi, Yashoswini Chakraborty, Revika Singh, Ibrahim B. Mokhtar, Aaron J. Kaat, Shaili Ganatra, Rachel O’Conor, Marlise Pierre-Wright

**Affiliations:** 1 Northwestern University Feinberg School of Medicine, Chicago, IL USA; 2 Department of Medicine, University of Illinois College of Medicine, Chicago, USA; 3 Department of Medicine, Northwestern University Feinberg School of Medicine, Chicago, IL USA; 4 Northwestern University, Evanston, IL USA

## Abstract

**Background:**

Trauma-informed care (TIC) acknowledges the events, experiences, and effects of trauma. Research shows that TIC may improve patient trust in providers, yet remains poorly integrated into graduate medical education.

**Objective:**

We conducted a needs assessment of internal medicine (IM) residents to (1) quantify knowledge, attitudes, perceived competence, practices, and barriers; (2) explore experiences with TIC; (3) identify factors shaping TIC implementation; and (4) determine unmet needs.

**Design:**

We conducted a multi-phase study involving a survey and a focus group. Survey data analysis included descriptive statistics, composite scores, Spearman’s correlations, and ANOVAs. Qualitative data analysis used an inductive and deductive thematic approach.

**Participants:**

We surveyed 69 IM residents at a large urban academic medical center (56% response rate). Ten residents participated in a focus group.

**Main Measures:**

The Trauma-Informed Care Provider Survey assessed TIC knowledge, opinions, perceived competence, barriers, and practices. The focus group explored experiences with trauma disclosures, barriers, facilitators, and training needs.

**Key Results:**

Residents demonstrated moderate knowledge (74%) and favorable opinions (80%) of TIC, but low self-rated competence (42%). Time constraints and lack of training were the most common barriers. Residents performed fewer than half of TIC practices on average; self-rated competence (*ρ* = 0.42, *p* = 0.0003) and attitudes (*ρ* = 0.33, *p* = 0.005) were positively associated, while lack of training predicted lower practice (*F* = 5.81, *p* = 0.005). Focus group themes include (1) residents understand TIC’s impact; (2) barriers hinder trauma screening; (3) residents feel unprepared to address trauma disclosures; (4) continuity of care is crucial for TIC; and (5) residents desire improved training.

**Conclusions:**

IM residents recognize TIC’s role in strengthening therapeutic relationships, yet multiple factors constrain consistent implementation. Addressing these barriers through improved training, clinical frameworks, and organizational support is essential to improving residents’ capacity to deliver patient-centered, trust-building care.

**Supplementary Information:**

The online version contains supplementary material available at 10.1007/s11606-026-10260-6.

## INTRODUCTION

Trauma refers to experiences that are perceived as physically or emotionally harmful and that have lasting impact on mental, physical, social, emotional, and spiritual well-being^1^. Traumatic events have been associated with chronic health conditions, mental health challenges, increased healthcare utilization, and reduced life expectancy^2–5^. Adverse childhood experiences (ACEs), such as abuse, neglect, and household dysfunction, represent a category of traumatic events that have been consistently associated with chronic health conditions, mental health challenges, increased healthcare utilization, and reduced life expectancy^2–5^. Furthermore, by disrupting an individual’s sense of safety and trust, trauma can reshape their self-perception and worldview, which can greatly impact clinical encounters^6^. Therefore, it is critical that clinicians understand and address the profound effects of trauma on health and patient healthcare experiences^6^.

Trauma-informed care (TIC) is an approach to healthcare that acknowledges the events, experiences, and effects of trauma on an individual and incorporates this understanding into healthcare practice^1^. Modern TIC originated with the work of Harris and Fallot in 2001who first articulated a “trauma-informed service system,” which reconceptualized trauma as a public health issue and proposed that service systems should be organized around an understanding of its widespread impact^7^. TIC is guided by the Substance Abuse and Mental Health Services Administration (SAMSHA) framework of the 4Rs: realizing trauma’s effects, recognizing the signs of trauma, responding to trauma, and resisting re-traumatization^1^. SAMHSA also outlines six guiding principles for TIC: safety, trustworthiness and transparency, peer support, collaboration and mutuality, empowerment voice and choice, and attention to cultural, historical, and gender issues^1^. This approach aims to foster trusting relationships between patients and healthcare providers, with the potential to improve patient outcomes^8,9^.

Internal medicine (IM) residents provide longitudinal, comprehensive care for diverse patient populations, many of whom have experienced trauma including ACEs, intimate partner violence, structural and historical trauma, and medical trauma related to hospitalization or invasive procedures^10–16^. However, many IM residents feel unprepared to address trauma effectively^10–12^. TIC curricula have been developed for various healthcare professions (including pediatric, emergency medicine, nursing, and primary care providers)^17–22^. However, existing curricula are limited and have not been designed specifically for IM residents or informed by resident-identified needs or the unique challenges IM trainees face^10,11,21^. To address this gap, we conducted a needs assessment to (1) quantify knowledge, attitudes, perceived competence, current practices, and barriers related to TIC; (2) explore residents’ experiences responding to trauma disclosures and caring for patients with trauma histories; (3) identify individual, clinical, and organizational factors that shape residents’ ability to provide TIC and build trust with patients; and (4) synthesize these findings to determine unmet needs and guide improvements in training and support for TIC practice.

## METHODS

### Design

We conducted a two-phase study with both quantitative and qualitative aspects. In Phase 1, we utilized a quantitative survey to assess IM residents’ knowledge, attitudes, perceived competence, practices, and barriers related to TIC in order to identify areas of underpreparedness and patterns associated with limited implementation. In Phase 2, we conducted a qualitative focus group to explore IM residents’ experiences with patients who have trauma histories, including facilitators and barriers to responding to trauma disclosures and the impact of these factors on building trust and delivering effective care, in order to identify key themes and unmet needs in TIC practice. The study employed a multiple-methods approach inspired by the sequential mixed-methods model^23^. We pulled from two different designs of mixed-methods studies^23^. Our sequence of collecting quantitative data followed by qualitative data was aligned with an explanatory design; however, the first phase did not inform the second^24^. Our assignment of equal priority to both phases aligned with the Convergent Design; however, the collection did not occur simultaneously^24^. By using a modified approach, we were able to use the high reliability and breadth of the quantitative survey and high validity and contextual depth of the qualitative phase separately to mutually enhance the overall findings. The Northwestern University Institutional Review Board approved all study protocols and procedures, and all participants provided written informed consent.

For both phases of the study, all categorical IM residents in the McGaw Medical Center of Northwestern University program were eligible to participate. Preliminary interns who proceed to another specialty after a year of internal medicine training were not included, as their training needs may differ from those of categorical IM residents. Categorical IM residents in this study primarily train in inpatient settings, including general medicine wards, intensive care units, and subspecialty consult services, with additional experience in outpatient continuity clinics. These residents work primarily at a large academic hospital site but also spend a portion of their time at a Department of Veteran Affairs (VA) hospital. In both phases, participants were recruited through a multifaceted strategy to optimize response rates including multiple emails, announcements during academic sessions, flyers in resident areas, and in-person outreach in resident workrooms. This strategy is consistent with best practices for improving physician participation^25–27^.

### Phase 1: Quantitative Survey

#### Recruitment and Data Collection

Survey recruitment occurred from December 2023 to March 2024. Participants provided electronic informed consent within the Qualtrics platform prior to completing the anonymous Trauma-Informed Care Provider Survey, which was developed by the Center for Pediatric Traumatic Stress^28^. We utilized the published survey version intended for healthcare providers caring for adult patients (the “all patients” version). This validated instrument includes 48 Likert scale–type items across five sections: (1) knowledge of TIC principles: 13 items on a 4-point Likert scale (1 = strongly disagree to 4 = strongly agree), (2) opinions about TIC: 7 items on the same 4-point Likert scale, (3) self-rated competence in TIC: 12 items on a 3-point Likert scale (1 = not competent to 3 = very competent), (4) perceived barriers to TIC implementation: 9 items on a 3-point Likert scale (1 = not a barrier to 3 = significant barrier), and (5) TIC practice performed in the past 6 months: 9 items on a binary scale (yes or no). Participants received a gift card upon survey completion.

#### Statistical Analysis

Survey data was analyzed using descriptive statistics. Composite scores were calculated based on the scoring rubric provided by the survey for knowledge (range 13–52), opinions favorable to TIC score (range 7–28), and self-rated competence (range 0–24), with higher scores indicating greater agreement or competence^28^. Four items were reverse coded to maintain scoring consistency^28^. Scores were reported as the average percentage of items answered in accordance with TIC principles, consistent with the standard approach used when reporting results for this survey^28^. We calculated means and standard deviations for each barrier and frequency of TIC practice. We performed Spearman’s rank-order correlations to determine whether residents’ knowledge, favorable opinions, or self-rated competence were associated with higher reported use of TIC practices. We reported both unadjusted and winsorized values to adjust for outliers and inconsistent category utilization. To evaluate the impact of specific perceived barriers on TIC practice, we conducted one-way analysis of variance (ANOVA) models. These assessed whether the mean number of TIC practices implemented differed significantly based on how participants rated specific barriers (i.e., “not a barrier,” “somewhat a barrier,” or a “significant barrier”).

### Phase 2: Focus Group

#### Recruitment and Data Collection

We conducted a 1-hour in-person focus group on February 6, 2024, with ten categorical IM residents. The final group consisted of the residents who were willing and able to attend the in-person session. The decision to conduct one focus group reflected challenges in recruiting busy residents despite repeated outreach efforts; however, methodological guidance indicates that an initial focus group typically identifies the majority of new issues and establishes code categories^29^. The objective of this phase was therefore to maximize code identification and explore preliminary themes. Residents received a gift card for their participation.

The focus group was facilitated by two investigators (M.P, U.T), who were colleagues of the participants with no supervisory role, using a semi-structured guide developed using insights from literature review and input from the authorship team (M.P, M.J) (Appendix A)^2,10–12,30–32^. The session was audio-recorded using a voice-activated recording device and transcribed verbatim using the Microsoft Word audio-to-text transcriber. U.T reviewed and edited the transcript for accuracy and formatting clarity. Participants identified themselves by assigned numbers to maintain confidentiality.

#### Analysis

The authors used a dualistic inductive and deductive thematic analysis to qualitatively analyze the data that was used^33^. For the deductive component, the authors created a preliminary codebook (Appendix B) based on the focus group guide^33^. Three investigators (U.T, M.P, Y.C) independently coded the transcript. Using an inductive approach of thematic analysis, the authors iteratively refined the codebook based on group discussions and completed three total rounds of coding until consensus was reached^33^. Dedoose software was used to conduct all qualitative analysis^34^.

## RESULTS

### Phase 1: Quantitative Survey

The survey was distributed to 113 categorical IM residents. 69 (61%) began the survey with 63 (56%) completing all questions. The baseline estimated response rate for an online survey among general health professionals is 38%; however, the use of monetary incentives and follow-up would make the likely response rate to range from approximately 38% to 66%, indicating our response rate is within expectations^26^. Data from participants who did not complete a given survey section were excluded from analysis for that given section. To reduce survey fatigue, demographic data was not collected.

#### Knowledge of Trauma-Informed Care

Residents demonstrated moderate knowledge of TIC, with a mean knowledge score of 38.9 (standard deviation (SD) 2.48) out of a possible 52 points (74%). The average scores on each individual item are shown in Appendix C. Less than half of residents (36%) correctly recognized that the severity of an injury or illness does not necessarily predict the intensity of an individual’s traumatic stress reaction. Only 26% of residents recognized that many individuals experiencing serious illness or injury will cope well on their own, and just over half (53%) of residents knew signs and symptoms of traumatic stress in ill or injured patients. Notably, 58% incorrectly believed that most individuals with life-threatening illnesses or injuries will develop significant posttraumatic stress response or disorder.

#### Opinions of Trauma-Informed Care

Residents expressed favorable opinions toward TIC, with a mean score of 22.4 (SD 2.29) out of 28 (80%). Individual items in the opinions section of the survey are shown in Appendix D. All respondents (100%) agreed that medical care should be made less stressful for patients, that providers can teach patients how to cope with trauma, that healthcare professionals should regularly assess for symptoms of traumatic stress, and that healthcare organizations should address how working with patients and families impacts staff.

#### Self-rated Competence in Providing Trauma-Informed Care

The average self-rated comfort and competence score of TIC was 10.1 (SD 3.76) of 24 possible points (42%). Table 1 shows that the majority of residents rated their skills in providing TIC as “not competent” or “somewhat competent.” Nearly half rated themselves as “not competent” in eliciting details of a traumatic event without re-traumatizing the patient (41%) or in changing or altering potentially traumatic hospital situations (47%). Additionally, half of residents felt “not competent” in providing basic trauma-focused interventions, while 53% lacked confidence in educating patients about common traumatic stress reactions and symptoms. In contrast, self-rated competence was highest in resident ability to respond calmly and without judgment to a patient’s emotional distress (42%).

**Table 1:** Internal Medicine Residents’ Self-rated Competence in Specific Aspects of Trauma-Informed Care (*N* = 66)

Specific aspects of trauma-informed care	Ratings (*N*, %)		
	Very competent	Somewhat competent	Not competent
Engaging with traumatized patients so that they feel comfortable talking to you/comforted by you	11 (17%)	47 (71%)	8 (12%)
Responding calmly and without judgment to a patient’s strong emotional distress	28 (42%)	35 (53%)	3 (5%)
Eliciting details of a traumatic event from a patient without re-traumatizing them	5 (8%)	34 (52%)	27 (41%)
Educating patients about common traumatic stress reactions and symptoms	4 (6%)	27 (41%)	35 (53%)
Changing or altering situations within the hospital that a patient might experience as traumatic	1 (2%)	34 (52%)	31 (47%)
Responding to a patient’s question about whether he/she will die	9 (14%)	49 (74%)	8 (12%)
Assessing a patient’s distress, emotional needs, and support systems soon after a traumatic event	7 (11%)	44 (67%)	15 (23%)
Providing basic trauma-focused interventions (assessing symptoms, normalizing, providing anticipatory guidance, coping assistance)	1 (2%)	32 (48%)	33 (50%)
Understanding how traumatic stress may present itself differently in patients of different ages, gender, or cultures	5 (8%)	34 (53%)	25 (39%)
Understanding the scientific or empirical basis behind assessment and intervention for traumatic stress	2 (3%)	28 (44%)	34 (53%)
Responding to colleagues’ distress, emotional needs, and need for support	15 (23%)	44 (69%)	5 (8%)
Managing your own work-related stress or distress	11 (17%)	50 (78%)	3 (5%)

#### Barriers to Implementing Trauma-Informed Care

**Figure 1:**
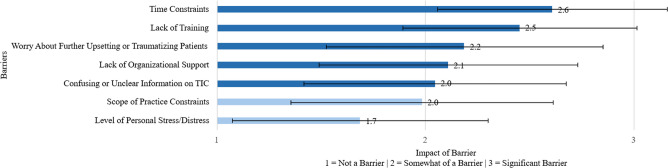
Internal medicine residents’ rating of factors that may be barriers to integration of trauma-informed care (*N* = 64).

Participants identified multiple barriers to administering basic trauma-informed assessments and intervention. The mean and standard deviation of scores for the barriers are described in Fig. 1. The most frequently reported barriers were time constraints (2.6 SD 0.55), lack of training (2.5 SD 0.56), and fear of re-traumatization (2.2 SD 0.66).

#### Implementing Trauma-Informed Practice

Residents endorsed implementing an average of 5 of 9 trauma-informed practices in the past 6 months (Table 2). The most frequently endorsed practices were encouraging patients to utilize their social support system (97%), assessing and caring for one’s own emotional and physical health (90%), and utilizing available support for oneself and one’s team (75%). Fewer than one-quarter of residents reported teaching families what to say to their family member after a difficult experience (22%) or providing information to the family about emotional or behavioral reactions that may indicate a need for help (24%).

**Table 2:** Internal Medicine Residents’ Report of Specific Trauma-Informed Practices Performed in the Past 6 Months (*N* = 63)

Specific trauma-informed practice	Have done this in past 6 months (*N*, %)
Ask a patient questions to assess his/her symptoms of distress	43 (68%)
Ask patients’ family members questions to assess their symptoms of distress	41 (65%)
Teach a patient specific ways to manage pain and anxiety during a procedure	29 (46%)
Teach a patient specific ways to cope with upsetting experiences	22 (35%)
Encourage patients to make use of their own social support system (family, friends, etc.)	61 (97%)
Teach family what to say to their family member after a difficult/painful/scary experience	14 (22%)
Provide information to family about emotional or behavioral reactions that indicate their family member may need help	15 (24%)
Assess and care for your personal emotional and physical health	57 (90%)
Utilize support for yourself/your team available from your organization	47 (75%)

Spearman’s correlation analyses (Table 3) revealed that self-rated competence was moderately and significantly correlated with practice (*ρ* = 0.42, *p* = 0.0003). Favorable opinions about TIC also showed a significant positive correlation (*ρ* = 0.33, *p* = 0.005). However, knowledge and perceived barriers were not significantly associated with TIC practice in either the original or revised models, to better meet statistical assumptions.

**Table 3:** Spearman’s Correlations Between Recent TIC Practices and Other Survey Sections

Variable	Original coding		Revised predictors and outcome	
	Rho	*p*-value	Rho	*p*-value
Knowledge	0.17	> 0.10	0.17	> 0.10
Opinions	0.33	0.005	0.33	0.005
Barriers	0.05	> 0.10	0.05	> 0.10
Self-rated competence	0.42	0.0003	0.42	0.0003

One-way ANOVA analyses (Table 4) indicated that among the perceived barriers, only “lack of training” was significantly associated with reduced use of TIC practices (*F* = 5.81, *p* = 0.005). While not statistically significant, “worry about further upsetting or retraumatizing patients” approached significance (*F* = 2.93, *p* = 0.06). In addition, the authors noted a trend toward fewer TIC practice implementations among those who identified “lack of organizational support” as a barrier.

**Table 4:** ANOVA for Relationship Between Barriers and Recent TIC Practices

Scale	Mean (SD)			MSE	*F*-statistic	*p*-value
	Not a barrier	Somewhat of a barrier	Significant barriers			
Time constraints	3 (2.8)	3.9 (7.8)	3.5 (1.8)	1.4	0.42	0.66
Scope of practice constraints	3.7 (2.1)	3.7 (1.8)	3.5 (1.7)	0.1	0.03	0.97
Lack of training	6.5 (0.7)	4.1 (2.0)	3.0 (1.3)	16.9	5.81	0.005
Confusing or unclear information on trauma informed care	3.5 (2.0)	3.8 (2.0)	3.5 (1.0)	0.6	0.17	0.85
Worry about further upsetting or traumatizing patients	4.9 (2.2)	3.3 (1.6)	3.8 (1.7)	9.2	2.93	0.06
Lack of organizational support	4.4 (1.9)	3.7 (1.9)	3.1 (1.3)	4.5	1.38	0.26
Level of personal stress/distress	3.5 (1.6)	3.8 (2.0)	3.2 (1.6)	1.5	0.47	0.63

### Phase 2: Focus Group

A total of ten IM residents (three PGY1s, three PGY2s, and four PGY3s) participated in the focus group, of which six were female and four were male. Gender and PGY level were self-reported by participants.

#### Thematic Analysis

Thematic analysis revealed five major themes related to residents’ experiences with TIC: (1) residents are aware of trauma and understand its impact on trust and patient care; (2) residents endorse multiple barriers to screening patients’ trauma histories during clinical encounters; (3) residents do not feel prepared to effectively respond to unprompted patient trauma disclosures; (4) continuity of care is essential for patients with trauma histories; and (5) residents express a strong desire for enhanced TIC training and frameworks to guide clinical encounters with patients with trauma histories. In addition to a description of each theme below, supplemental quotes for themes are presented in Table 5.

**Table 5:** Qualitative Results of Focus Group

Theme	Representative focus group quotes
Residents are aware of trauma and understand its impact on trust and patient care	“I think you know [trauma] is all too common and I think it’s really important to normalize it.” (#1, PGY3)
Residents endorse multiple barriers to screening patients’ trauma histories during clinical encounters	“… you often just don’t have the time to spend… 20–30 min… to have that type of rapport with the patient especially when the situations you’re talking about are often pretty serious.” (#7, PGY3) “I don’t want to go digging for something that might inadvertently bring up trauma and trigger somebody.” (#3, PGY1)
Residents do not feel prepared to effectively respond to unprompted patient trauma disclosures	“Am I going to say the wrong thing? Am I going to come across as insensitive? Am I going to come across as meaning well, but saying something that’s totally, you know, counterproductive to what they want to hear.” (#5, PGY2)
Continuity of care is essential for patients with trauma histories	“I was going to move on within the next year… some patients… who I’m just getting to know, it feels a lot harder to catch up on everything. And then the patients… have the same sense that if they know you’re not going to see them for more than a couple of visits, they… may not feel as invested.” (#2, PGY3)
Residents express a strong desire for enhanced TIC training and frameworks to guide clinical encounters with patients with trauma histories	“I think training experience is a big part of it too. It’s kind of like a vicious cycle where if you never see it, you don’t know what to ask, and then when you are confronted with it, you feel kind of scared because you don’t have the proper training and you don’t feel like you have the proper tools.” (#1, PGY3)

##### Residents Are Aware of Trauma and Understand Its Impact on Trust and Patient Care

Participants demonstrated a strong understanding of TIC and its relevance to patients’ interactions within the healthcare system. Residents acknowledged that past trauma, especially within a medical context, could lead to mistrust.

“I feel like I see trauma a lot in the form of mistrust in healthcare. I see a lot of patients who feel distrustful of their healthcare team or the hospital in general because of past traumas...they felt like their care was mismanaged in the past” (#8, PGY1).

Residents also highlighted positive outcomes when trauma is addressed effectively. One participant suggested that sensitive and appropriate handling of trauma can foster trust.

“You know, as a system and as a clinician, I think if you address the trauma well, it can often be a major boost in terms of trust” (#2, PGY3).

##### Residents Endorse Multiple Barriers to Screening Patients’ Trauma Histories During Clinical Encounters

Residents identified several barriers that hindered their ability to screen for trauma. Time was a significant barrier, especially in outpatient settings.

“On the outpatient side, the biggest constraint is going to be time if the patient is coming in for other medical issues. Oftentimes, even though the traumatic issues are just as important and can make just as big of an impact on their well-being, you often just don’t have the time to spend” (#7, PGY3).

They also expressed a lack of confidence in responding to a history of trauma due to insufficient training. One participant described it as a “vicious cycle” where limited exposure to TIC leads to uncertainty about what to ask and a fear of causing harm, especially when they have not been trained in how to navigate those situations (#1, PGY3). This went hand in hand with anxieties about potentially re-traumatizing patients by inquiring about their trauma history.

“Often I feel like I... might be upsetting the patient by bringing up something that might not seem relevant to their care, which might make them upset... and maybe triggering them or asking questions that might not have anything to do with the reason why they’re hospitalized” (#6, PGY1).

Additional barriers to building trust and delivering comprehensive care for patients with trauma histories included multiple resident rotations and the absence of long-term patient relationships. This lack of continuity was perceived as potentially re-traumatizing, as patients often must recount their story “again, and again, and again” to new providers (#10, PGY2). Additionally, residents expressed concerns about breaking trust during care transitions, particularly when intimate trauma histories were shared second-hand with other clinicians, leaving patients to discover that “the team member has now heard [about the trauma] second-hand” (#4, PGY3).

##### Residents Do Not Feel Prepared to Effectively Respond to Unprompted Patient Trauma Disclosures

Although residents did not routinely screen for trauma, they reported that patients often disclosed trauma histories during routine care. Residents recounted instances in inpatient settings where patients spontaneously shared experiences of sexual or physical trauma (#5, PGY2). Others described situations where trauma was disclosed to team members like social workers or case managers (#1, PGY3). When asked about their comfort level engaging with these patients, one resident admitted feeling “pretty uncomfortable,” a sentiment shared by others who worried about saying the wrong thing, triggering patients, or appearing insensitive (#5, PGY2).

The residents’ discomfort was largely attributed to previously discussed barriers, including inadequate training and fears of inadvertently causing harm by “bringing up those emotions” without knowing how to help patients process them (#10, PGY2).

Residents also felt uncertain about how to proceed following trauma disclosures, citing a lack of clear protocols or guidelines.

“Another barrier is knowing next steps... digging into someone’s trauma history, but then being like... thank you for sharing, but social work will handle it next. I don’t... feel great about that” (#3, PGY1).

Residents also acknowledged frequently deferring to other healthcare professionals, such as social workers or psychologists, as they struggled to define their role in supporting patients who disclosed trauma.

“If we are to explore the trauma that a patient has, what is our end goal? Is our end goal to set them up with somebody else or is the end goal to own this issue and be that provider for the patient with respect to their trauma? I think that is pretty unclear” (#7, PGY3).

##### Continuity of Care Is Essential for Patients with Trauma Histories

The impact of care continuity was frequently discussed, including the importance of consistent relationships and the harm caused by frequent care transitions. Participants acknowledged the inherent challenges of the structure of residency training rotations.

“...some patients that have been following for a long time who I’m just getting to know, it feels a lot harder to catch up on everything. And then the patients also, I think, have the same sense that if they know you’re not going to see them for more than a couple of visits, I feel like the patients may not feel as invested as well” (#2, PGY3).

In terms of outside resources, residents recognized the need for referrals to mental health professionals, but they acknowledged a lack of systemic support for patients, contributing to feelings of helplessness and uncertainty.

“My concern is yes, if this patient is screening positive, it’s like a three month wait to get someone into a psych appointment... We screened them positive. We know they have trauma and then we just sit on it” (#10, PGY2).

They also highlighted instances where greater continuity of care led to positive outcomes for patients with trauma.

“I can definitely say at our... VA clinic, some of the patients had followed there for a long time and a few of them did have significant military related trauma that they felt like was treated really well... from their long time continuity with my attending in clinic felt like... a lot of trust in the system from how well the system handled their trauma and the support that the system was able to give.” (#2, PGY3)

##### Residents Express a Strong Desire for Enhanced TIC Training and Frameworks to Guide Clinical Encounters with Patients with Trauma Histories

Residents expressed dissatisfaction with the current state of TIC training, describing it as inadequate for preparing them to address trauma effectively in clinical encounters. They specifically emphasized the need for concrete frameworks to guide initiating conversations about trauma, responding to disclosures, and managing the complexities of providing TIC.

“... talking about code status and learning a very specific framework with specific language and the situations it was appropriate for. I still use that every single day and I feel like one, it standardizes things amongst the residents, and two, I feel like it goes a lot better when you have a more evidence-based approach than kind of coming up with something completely on your own” (#2, PGY3).

Residents expressed enthusiasm for training methods that use simulation and role-playing to practice incorporating TIC in a safe and controlled environment. There was also a desire for a more longitudinal approach to TIC training by integrating it throughout residency with recurring didactic sessions. Finally, residents indicated that it would be valuable to learn from clinicians experienced in working with patients with trauma histories.

“It may be helpful to have the residents meet with for example, one of the VA [Veteran Affairs] psychology providers...So they can just tell us. “Hey, this is sort of where I picked the conversation up and when somebody tells me that this happened to them, this is sort of the way that I react to that information and how I approach somebody who I’m meeting for the first time in site clinic that tells me this'” (#5, PGY2).

## DISCUSSION

This study contributes to the growing body of mixed-methods research examining barriers to resident physicians’ delivery of TIC and provides specific insights into mechanisms influencing the implementation of TIC practices among IM residents, including training gaps, fears of re-traumatization, and disrupted continuity of care^10,21,35,36^. Given that IM residents, by virtue of their role in the hospital and primary care, routinely care for adults with trauma histories, clinician competency in TIC is essential for fostering trust and supporting effective clinical care^10–16^. We found that while IM residents demonstrated favorable attitudes toward TIC, they exhibited moderate knowledge, low self-rated competence, and inconsistent delivery of TIC, aligning with prior research findings^10–12^.

Notably, our study shows that residents recognize the impact of trauma on patient outcomes, suggesting that existing medical education may be reinforcing awareness of trauma-informed principles^17,37^. Residents in our focus group also described instances where thoughtful engagement around trauma strengthened therapeutic alliances and demonstrated positive attitudes toward TIC. However, our residents in the survey scored training gaps, time constraints, and fear of re-traumatization as the most significant barriers. Residents who identified a lack of training as a barrier demonstrated significantly lower trauma-informed care practices, highlighting the meaningful real-world implications of this gap. The focus group further elucidated these findings with participants describing a cycle in which insufficient training produced discomfort with asking about trauma, heightening fears of re-traumatization, and limiting skill development opportunities to develop practical skills, ultimately affecting residents’ ability to establish a therapeutic relationship with these patients. Participants also felt uncertain about the next steps following a disclosure around a patient’s history of trauma, including how to validate patients’ emotions or facilitate appropriate referrals. These challenges have been described in prior studies examining gaps in IM TIC resident training and having direct implications for undermining the provider-patient relationship^10,12,21,38,39^. This reinforces the need for improved training on TIC, a modifiable target for enhancing residents’ ability to engage, validate, and build therapeutic relationships with patients with trauma histories.

Recent years have seen an increase in TIC curricula driven by several key initiatives to address this need. The Trauma-Informed Medical Education (TIME) framework proposed the integration of TIC principles into undergraduate and graduate medical education (GME) with an emphasis on trauma epidemiology, clinical skills, and self-care^37^. In 2022, the National Collaborative on Trauma-Informed Health Care Education and Research (TIHCER) released the first validated set of TIC competencies for undergraduate medical education to scaffold integration in medical schools^17^. A scoping review shows that most programs include didactic components, multidisciplinary teaching teams, and opportunities for trainees to practice skills through role play, simulation, or patient interviews^40^. While the Accreditation Council for Graduate Medical Education (ACGME) mandates that ethics, professionalism, and patient-centered care are incorporated into GME, there is currently no explicit requirement for TIC to be a part of the core curriculum^41^. Furthermore, existing curricula are often short, inconsistently implemented across specialties, and less often reinforced with real-world practice or system-level support^19,38,42,43^. Residents frequently report a conceptual understanding but insufficient practical competence, identifying a persistent training gap from medical school to residency^10–12^.

Additionally, residents in our focus groups identified care fragmentation as another barrier to providing TIC, which is an issue in our disjointed healthcare system and especially salient for IM residents in academic medical centers^44^. Frequent provider changes and uncoordinated inpatient to outpatient transitions limit relational continuity with residents^44^. Our residents described long-standing provider-patient relationships as facilitators to providing TIC, consistent with prior literature^8^. The tension our participants recognized between the value of continuity while practicing in inherently discontinuous training environments highlights a key challenge. Residency programs should teach TIC skills that are adaptable across both long-term care and acute care settings. Structural changes such as emphasizing warm handoffs, standardized communication tools, and collaborative interdisciplinary workflows may help mitigate some of these limitations of the IM residency structure^45^.

Residents in our study offered concrete ideas for strengthening TIC training, emphasizing the value of longitudinal, skills-based, and contextually relevant approaches, such as ongoing interactive workshops. Specifically, being trained using evidence-based frameworks on how to best navigate conversations around trauma. Participants also expressed a desire for training led by medical professionals who regularly work with patients with trauma histories, such as social workers and psychologists. These preferences align with growing evidence supporting experiential learning in psychologically safe environments and early, structured exposure to TIC helping to normalize it as a routine aspect of care, reduce discomfort, and minimize variation in implementation during residency^17,37,46–48^. Participants also acknowledged the emotional challenges of trauma-related encounters and the importance of attending to provider well-being. To address these needs, TIC trainings and institutional supports should also emphasize the importance of self-care, team support, and organizational responsibility to mitigate risks of burnout and secondary traumatic stress.^49^

### Limitations and Future Directions

Limitations to our study include that the study was conducted at a single, major academic institution located in an urban setting, which limits the generalizability of the findings. Although the institution serves a socioeconomically and racially diverse adult population and prior TIC studies have sampled predominantly academic medical environments, the experiences of the residents may differ from those in community hospitals, rural programs, or smaller academic centers^10,11^. Residents were recruited using a convenience sampling approach based on availability and interest in participation. Although multiple recruitment strategies were employed to maximize participation, some degree of selection bias was likely. While the survey response rate of 56% represents most eligible residents, it does not capture the perspectives of all residents. We conducted one focus group, which limits the breadth of perspectives captured; this decision reflected the difficulty of recruiting busy residents despite extensive outreach. Although the group generated substantial thematic and code development, conducting more focus groups would have allowed for a wider range of perspectives and thematic saturation. The study only included categorical IM residents, and the findings may not extend to residents in other specialties or disciplines. We were unable to assess how residents’ intended career paths (e.g., primary care, subspecialty training, hospital medicine) influenced their attitudes toward TIC or their decision to participate, which may have shaped both survey responses and focus group dynamics. We did not collect demographic information about participants. We did not assess how resident well-being, traumatic stress, or burnout may have affected engagement, though some participants alluded to emotional strain during trauma-related encounters. Future research should explore how resident characteristics and well-being intersect with TIC competence and implementation. The authors intentionally focused on residents’ perceptions of the impact of TIC and barriers to delivering this care on the therapeutic relationship and patient care, as these domains remain relatively understudied in the literature. Future studies should explore trainee experiences with these additional components of TIC such as universal precautions, psychological and physical safety practices, inclusive communication strategies, trauma-informed physical examination approaches, and peer support.

## CONCLUSION

This study highlights a critical gap between IM residents’ understanding of and positive attitudes toward TIC and their ability to implement it effectively. Addressing both individual and system-level barriers through targeted skills training, clearer clinical guidance, and stronger organizational support is essential to improving residents’ ability to provide TIC that fosters trust and strengthens the therapeutic relationship.

## Supplementary Information

Below is the link to the electronic supplementary material.

Supplementary file1 (DOCX 36.2 KB)

Supplementary file2 (DOCX 59.3 KB)

Supplementary file3 (DOCX 58.1 KB)

Supplementary file4 (DOCX 56.0 KB)

## Data Availability

All data generated or analyzed during this study are included in this published article and its supplementary information files. Any additional data that are not included may be available from the corresponding author on reasonable request.
