# Supplementary material for: Internal Medicine Residents’ Challenges in Trauma-Informed Care and Impact on Patient Care: A Multiple-Methods Study
Source: J Gen Intern Med. 2026 Mar 16;41(8):2141–51. doi: 10.1007/s11606-026-10260-6 (PMC13241347; doi:10.1007/s11606-026-10260-6)
Supplement: Supplementary file 1 — Supplementary file1 (DOCX 36.2 KB) [file 11606_2026_10260_MOESM1_ESM.docx]

**Appendix A**

Focus Group Guide:

Do you have experiences with patients who have disclosed histories of trauma? If so, did you ask for a trauma history or did the patient disclose on their own? How did you react?

What are some barriers to addressing a patient’s trauma?

How comfortable do you feel that you can engage with a patient’s trauma without re-traumatizing them?

If you do take a trauma history, what do you ask?

Did you feel prepared to address trauma with patients? Did you get training in trauma-informed care? If you received training, was it enough?

Describe whether you feel that patients who have trauma histories have more or less trust in the health care system? In you as a resident-physician? In your perspective, among patients with trauma histories, is there a difference in trust with attending vs resident-physicians?

How do you work to gain trust from your patients with trauma histories?

What resources do you draw on when your patient discloses a trauma history?

When would be the best time to address a history of trauma with a patient? When admitting the patient? During rounds? After rounds? On discharge?

How can we improve a resident’s ability to handle patients in a trauma informed manner?
