# Supplementary material for: Internal Medicine Residents’ Challenges in Trauma-Informed Care and Impact on Patient Care: A Multiple-Methods Study
Source: J Gen Intern Med. 2026 Mar 16;41(8):2141–51. doi: 10.1007/s11606-026-10260-6 (PMC13241347; doi:10.1007/s11606-026-10260-6)
Supplement: Supplementary file 2 — Supplementary file2 (DOCX 59.3 KB) [file 11606_2026_10260_MOESM2_ESM.docx]

**Appendix B**

| **Theme** | **Subtheme** | **Code Description** |
| --- | --- | --- |
| Barriers to TIC Implementation | Time Constraints | References to lack of dedicated time (e.g., in clinic, during rounds) preventing TIC implementation. |
|  | Lack of Training | Explicit statements about the absence, inadequacy, or deficiency of formal TIC education or mentorship in residency. |
|  | Resource Access | Difficulty knowing, referring, and using external resources to support patients. |
|  | Fear of Retraumatization | Resident hesitation regarding initiating trauma discussions due to worry about causing further patient distress or harm. |
|  | Stigma | Discussion of the negative beliefs, shame, or judgment (either patient-perceived or internal resident belief) surrounding mental illness or trauma history. |
|  | Resident Discomfort | Emotional or psychological unease experienced by the resident when confronted with trauma disclosures or providing TIC (e.g., feelings of inadequacy, awkwardness, or anxiety). |
| Trauma Identification | Screening for Trauma | Discussion of formal tools, checklists, or systematic processes used for routine trauma assessment. |
|  | Trauma History Taking | Methods used or in soliciting detailed information about past trauma. |
|  | Broaching the Subject of Trauma | Specific language, techniques, or difficulty introducing the topic of trauma history in a sensitive manner. |
|  | Trauma Disclosure | Instances where patients spontaneously shared, or were prompted to share, their trauma history with the resident or team. |
| Trauma Management | Next Steps After Trauma Disclosures | Actions, therapeutic responses, or perceived responsibilities taken by the resident immediately after a patient discloses trauma. |
|  | TIC Practices | General application of trauma-informed practices. |
|  | Communication | Focus on the use of trauma-sensitive language, non-judgemental tone, and effective de-escalation strategies during patient interaction. |
|  | Documentation | Challenges or suggestions regarding the appropriate, safe, and sensitive recording of trauma history in the patient chart. |
| Continuity of Care | Resident Relationship with Continuity of Care | Discussion of residency rotation structure, short time on service, or frequent transitions preventing the resident from developing long-term relationships. |
|  | Continuity of Care with Patients with Trauma Histories | Statements highlighting the patient's desire for consistent providers, the difficulty of building trust, or the potential for re-traumatization when forced to repeatedly recount their history to new team members. |
| Patient Trust | Patient Trust in Residents | Patient trust in the competency and reliability of the individual resident. |
|  | Patient Trust in Healthcare | Patient trust in the healthcare system, hospital, or organization due to past traumatic experiences. |
| Healthcare Setting | Inpatient | Experiences specific to the inpatient setting. |
|  | Outpatient | Experiences specific to the outpatient setting |
|  | VA | Experiences specific to the Veterans Affair Hospital/Clinic setting. |
|  | ICU | Experiences specific to the ICU setting. |
| Healthcare Team Members | Residents | Discussion of the specific role, responsibilities, or challenges faced by residents. |
|  | Social Workers | Discussion of the specific role, responsibilities, or challenges faced by social workers. |
|  | Nurses | Discussion of the specific role, responsibilities, or challenges faced by nurses. |
|  | Psychologists | Discussion of the specific role, responsibilities, or challenges faced by psychologists. |
|  | Chaplains | Discussion of the specific role, responsibilities, or challenges faced by chaplains. |
| Types of Trauma | Medical Trauma | Trauma stemming from the healthcare system itself (e.g., previous poor care, painful procedures, institutional neglect). |
|  | Sexual Trauma | References to experiences of sexual abuse or assault. |
|  | Military Trauma | Trauma stemming from military service, combat, or deployment. |
|  | Secondary Trauma | Discussion of vicarious trauma, burnout, or emotional distress experienced by the resident. |
|  | Marginalized Communities | Discussion of the impact of trauma related to systemic oppression, cultural issues, or experiences as part of marginalized communities. |
| Resident Suggestions for Improvement | Resident Desires for TIC Frameworks | Specific requests for standardized, evidence-based training frameworks to guide asking, responding, and taking action related to trauma. |
|  | Residents Desires for TIC Training | Suggestions for resident TIC curriculum delivery (e.g. simulation, role-playing, longitudinal sessions). |
|  | Support for Healthcare Workers | Recommendations for organizational systems to address resident stress, distress, and support for healthcare workers providing TIC. |
