# Supplementary material for: Internal Medicine Residents’ Challenges in Trauma-Informed Care and Impact on Patient Care: A Multiple-Methods Study
Source: J Gen Intern Med. 2026 Mar 16;41(8):2141–51. doi: 10.1007/s11606-026-10260-6 (PMC13241347; doi:10.1007/s11606-026-10260-6)
Supplement: Supplementary file 3 — Supplementary file3 (DOCX 58.1 KB) [file 11606_2026_10260_MOESM3_ESM.docx]

**Appendix C**

Supplementary Table 1. Internal medicine residents’ knowledge regarding trauma informed care

| Knowledge Items | Correct Responses: Agree/Strongly Agree* (N, %) |
| --- | --- |
| *Prevalence, risk factors, and course (N = 67)* | |
| Almost everyone who is seriously injured or ill has at least one traumatic stress reaction in the immediate aftermath of the event | 63 (94%) |
| It is inevitable that most individuals who experience a life-threatening illness or injury will go on to develop significant posttraumatic stress or PTSD* | 39 (58%) |
| Individuals who are more severely injured or ill generally have more serious traumatic stress reactions than those who are less severely injured or ill* | 24 (36%) |
| Individuals who, at some point during the traumatic event, believe that they might die are at greater risk for posttraumatic stress reactions | 63 (94%) |
| Many individuals cope well on their own after experiencing serious illness or injury | 18 (26%) |
| *Signs and symptoms (N = 66)* | |
| The psychological effects of an injury or illness often last longer than the physical symptoms | 65 (98%) |
| Individuals with significant posttraumatic stress reactions usually show obvious signs of distress* | 56 (85%) |
| I know the common signs and symptoms of traumatic stress in ill or injured patients | 35 (53%) |
| Some early traumatic stress reactions in patients can be part of a healthy emotional recovery process | 63 (95%) |
| *Effectiveness of screening and intervention (N = 66)* | |
| There are things that providers can do to help prevent longer-term posttraumatic stress in ill and injured patients | 65 (98%) |
| There are effective screening measures for assessing traumatic stress that providers can use in practice | 51 (77%) |
| Healthcare staff can themselves experience signs of physical and/or emotional distress related to their work | 66 (100%) |
| The risk for staff distress is strongly influenced by both personal and work-place factors | 66 (100%) |

*****Note. For items 2, 3, and 7, “disagree/strongly disagree” represents a correct response.
