# Supplementary material for: Internal Medicine Residents’ Challenges in Trauma-Informed Care and Impact on Patient Care: A Multiple-Methods Study
Source: J Gen Intern Med. 2026 Mar 16;41(8):2141–51. doi: 10.1007/s11606-026-10260-6 (PMC13241347; doi:10.1007/s11606-026-10260-6)
Supplement: Supplementary file 4 — Supplementary file4 (DOCX 56.0 KB) [file 11606_2026_10260_MOESM4_ESM.docx]

**Appendix D**

Supplementary Table 2. Internal medicine residents’ opinions regarding trauma-informed care (N = 66)

| Opinions | Correct Responses: Agree/Strongly Agree (N/__; %) |
| --- | --- |
| Providers should focus on medical care for hospitalized patients as opposed to patients’ mental health* | 60 (91%) |
| The way that medical care is provided can be changed to make it less stressful for patients | 66 (100%) |
| Providers can teach patients how to cope with trauma | 66 (100%) |
| Health care professionals should regularly assess for symptoms of traumatic stress | 66 (100%) |
| It is necessary for providers to have mental health information about their patients in order to provide appropriate medical care | 57 (86%) |
| I have colleagues I can turn to for help with a patient experiencing significant traumatic stress | 47 (71%) |
| Healthcare organizations should address how working with patients and families impacts staff | 66 (100%) |

*Note. For item 1, “disagree/strongly disagree” represents an opinion favorable to trauma-informed care).
